# Construction of the indicator-free electrochemical biosensor with magnetically self-assembly based on Fe_3_O_4_/α-Fe_2_O_3_ magnetic heterogeneous nanorods for the ultra-sensitive detection of CYFRA 21-1 DNA

**DOI:** 10.3389/fchem.2025.1696542

**Published:** 2025-12-12

**Authors:** Xiaoting Yi, Peng Deng, Zhou Wang, Jiawei Wang, Hongfei Wang, Zhan-ao Wu

**Affiliations:** 1 Zhenjiang 359 Hospital, Zhenjiang, China; 2 The People’s Hospital of Danyang, Affiliated Danyang Hospital of Nantong University, Zhenjiang, China; 3 College of Vanadium and Titanium, Panzhihua University, Panzhihua, China; 4 Zhucheng Maternal and Child Health Hospital, Zhucheng, China

**Keywords:** aptamer, CYFRA 21-1 DNA, detection, electrochemical biosensor, Fe3O4/α-Fe2O3@Au magnetic nanocomposites

## Abstract

**Introduction:**

Lung cancer prevalence rate has been rising steadily in recent years, for the prevention and treatment, the detection of tumor marker CYFRA 21-1 DNA demonstrates its significance.

**Methods:**

In this work, an electrochemical biosensor was constructed for sensitive detection of CYFRA 21-1 DNA based on the novel developed Fe_3_O_4_/α-Fe_2_O_3_ magnetic heterogeneous nanorods (MHNRs). Firstly, Fe_3_O_4_/α-Fe_2_O_3_ MHNRs were prepared by hydrothermal-calcination method, and then Fe_3_O_4_/α-Fe_2_O_3_@Au magnetic nanocomposites (MNCs) were obtained though gold-coating. Subsequently, the magnetic self-assembling electrochemical biosensor based on Fe_3_O_4_/α-Fe_2_O_3_@Au MNCs was successfully constructed, which was verified by cyclic voltammetry (CV) and electrochemical impedance spectroscopy (EIS). To optimize the biosensor’s experimental conditions and evaluate its performance, differential pulse voltammetry (DPV) was conducted.

**Results and Discussion:**

The results showed that the detection range of CYFRA 21-1 DNA was 10 pM–10 μM, the limit of detection (LOD) was 1.5 pM. The biosensor exhibited excellent selectivity, reproducibility, and stability; the relative standard deviation (RSD) was 2.01%. The average recovery rate in the spiked diluted human serum samples was 101.4%, and the RSD was ≤5.2%, indicating that the biosensor possessed promising prospect.

## 1 Introduction

Based on the statistics of the International Agency for Research on Cancer (IARC), approximately one fifth of the global human population will be diagnosed with cancer during their lifetime, there into, lung cancer is the most frequently malignant tumor with an incidence of 12.4% and mortality of 18.7% (Bray et al., 2024). The early-stage symptoms of lung cancer are not obvious and non-specific, which mimic those of other respiratory diseases or common colds (Candal-Pedreira et al., 2024; Tsiligianni et al., 2024). Therefore, the majority of lung cancer cases are diagnosed at an advanced stage, thus missing the best treatment period (Servadio et al., 2024). While, early diagnosis can effectively improve the survival rate of patients (Basha et al., 2025; Blanco-Villar et al., 2024; Tesema et al., 2025). Consequently, the development of a real-time and accessible modality for the early lung cancer detection represents an urgent need (Fang et al., 2025; Wang Y. Z. et al., 2025).

Minimally invasive and non-invasive body fluid detection is a novel method for dynamic monitoring of cancer development, offering considerable advantages in terms of safety and comfort (Huang S. et al., 2024). Serum detection of biomarkers is a powerful means to promote prevention, diagnosis, treatment, and prognosis of tumors. The biomarkers with diagnostic value for lung cancer mainly include cytokeratin 19 fragment (CYFRA 21-1) (Wang et al., 2020), neuron-specific enolase (NSE) (Wang K. Y. et al., 2025), and carcinoembryonic antigen (CEA) (Chen, 2025), etc. It is worth noting that non-small cell lung cancers accounts for the significant majority of lung cancer patients (Li et al., 2021), and CYFRA 21-1 DNA has been verified as a reliable diagnostic biomarker for NSCLC (Zhao et al., 2020). Thus, the detection of serum CYFRA 21-1 DNA concentration is particularly important for the clinical diagnosis of early-stage lung cancer. So far, these several techniques including PCR (Mishra et al., 2018), polysaccharide and eATRP dual amplification sensor (Zhao et al., 2020), LoC-SERS (Bao et al., 2023), and SPR (Wang et al., 2016) have been successfully employed for the sensitive detection of CYFRA 21-1 DNA. However, there still exhibits several limitations, including but not limited to complex instruments, cumbersome operation, long response time and low economic efficiency. Nowadays, the detection of biomarkers is evolving toward convenient, rapid, and cost-effective strategies, with electrochemical biosensors emerging as a particularly promising solution.

In recent years, biosensors for biomarkers detection utilizing magnetic nanomaterials have gained widespread application due to their unique advantages such as magnetic separation and magnetic immobilization (Farag et al., 2025; Wang et al., 2024; Yue et al., 2025). Magnetic ferrite nanomaterials, particularly α-Fe_2_O_3_ (hematite) and Fe_3_O_4_ (magnetite) have been extensively applied in biosensing owing to the characteristics of exceptional biocompatibility, low toxicity, and high surface area (Alshammari et al., 2024; Da Silva and Brett, 2020; Xue et al., 2025). Furthermore, integration with magnetic glassy carbon electrodes (MGCE) enables full utilization of the magnetic nanomaterials’ inherent advantages, significantly simplifying experimental procedures (Su et al., 2025; Xu et al., 2025). However, the magnetic property of α-Fe_2_O_3_ magnetic nanomaterials is too weak to enable the immobilization process (Tombuloglu et al., 2020), while that of Fe_3_O_4_ magnetic nanomaterials is too strong (Lu et al., 2025), causing the problem of agglomeration. To obtain the appropriate magnetic property for wider application, some scholars have developed Fe_3_O_4_/α-Fe_2_O_3_ magnetic heterogeneous nanomaterials (Liu et al., 2022; Wang et al., 2018). Moreover, the introduction of AuNPs has architected Fe_3_O_4_/α-Fe_2_O_3_@Au magnetic nanocomposites, which not only enables superior current amplification efficiency in working electrodes but also creates the foundation for biosensor construction (Fukuzumi et al., 2025; Miao et al., 2025; Munusamy et al., 2025).

In this project, an electrochemical biosensor was developed employing Fe_3_O_4_/α-Fe_2_O_3_ magnetic heterogeneous nanorods (MHNRs) as base substrate, where AuNP-ssDNA conjugate form a high-affinity bond as the connection that enable ssDNA to specifically capture CYFRA 21-1 DNA based on base complementary pairing for target detection. This system undergone magnetic self-assembly onto the MGCE surface, with subsequent microscopic events transformed into visual electrical signals through interface electron transfer kinetics. In particular, the biosensor was defined as indicator-free because it dispensed with the conjugation of signal-reporting moieties (e.g., fluorophores or electroactive molecules) to the target. Electrochemical characterization techniques, cyclic voltammetry (CV) and electrochemical impedance spectroscopy (EIS) were employed for the biosensor fabrication and interfacial analysis, while differential pulse voltammetry (DPV), owing to superior sensitivity, is utilized for parameters optimization and analytical performance evaluation.

## 2 Experimental

### 2.1 Materials

The materials, reagents and the corresponding suppliers are provided in the Supplementary Material.

The sequences of single-stranded oligonucleotides were followed.

Sulfhydryl probe (ssDNA): 5′-SH-GAAGGGAGGAATGGTGT​CAGGGGCG-3′

CYFRA 21-1 DNA (tDNA): 5′-CGC​CCC​TGA​CAC​CAT​TCC​TCC​CTT​C-3′

Single-base mismatch sequence (SBM DNA): 5′-CGC​CCC​TGA​CTC​CAT​TCC​TCC​CTT​C-3′

Double-base mismatch sequence (DBM DNA): 5′-CGC​GCC​TGA​CTC​CAT​TCC​TCC​CAT​C-3′

Negative control sequence (NC DNA): 5′-TAT​TAG​CCG​TCA​GTG​GAA​AGG​ACC​T-3′.

### 2.2 Preparation and characterization of Fe_3_O_4_/α-Fe_2_O_3_@Au MNCs

The Fe_3_O_4_/α-Fe_2_O_3_ MHNRs were fabricated using a hydrothermal-calcination method. Initially, 2.0 g of anhydrous ferric chloride (FeCl_3_) and 1.0 g of polyvinylpyrrolidone (PVP) were dissolved in 70 mL double-distilled water (DDW). The system was then subjected to ensure complete dissolution and homogeneity. After transferring into a Teflon-lined stainless-steel autoclave, it was placed in hydrothermal treatment at 100 °C for 10 h in a programmable temperature-controlled furnace. After the reaction, the supernatant was removed to get the sediment, which was subsequently washed three times with anhydrous ethanol, and then dried to obtain β-FeOOH nanorods.

Next, galactose and β-FeOOH nanorods were uniformly mixed in a 2:1 mass ratio and transferred into a crucible for further processing. It was calcined in a muffle furnace at 300 °C for 30 min, the calcined product was Fe_3_O_4_/α-Fe_2_O_3_ MHNRs.

To achieve Fe_3_O_4_/α-Fe_2_O_3_@Au MNCs, the preparation of Fe_3_O_4_/α-Fe_2_O_3_@PEI was a prerequisite. 1.6 g of polyvinylimine (PEI) was added in 160 mL of DDW, stirring thoroughly until fully dissolved. The solution was supplemented with 60 mg of Fe_3_O_4_/α-Fe_2_O_3_ MHNRs, and the resulting suspension was maintained at ultrasound for 30 min. The suspension was transferred to a round-bottom flask and reacted in a water bath at 90 °C for 90 min with magnetic stirring. Upon completion of the reaction, the precipitate was collected by centrifugation. It was subsequently washed twice with DDW and dried to obtain Fe_3_O_4_/α-Fe_2_O_3_@PEI.

The Fe_3_O_4_/α-Fe_2_O_3_@Au MNCs were prepared via the modified sodium borohydride reduction method for chloroauric acid. The whole experimental procedures were conducted under low-temperature conditions. 15 mg Fe_3_O_4_/α-Fe_2_O_3_@PEI were dispersed in 225 mL of DDW under ultrasound treatment for 10 min 1.5 mL chlorauric acid solution (20 mg/mL) was added dropwise to the suspension under continuous stirring. The mixture was then kept under ultrasound for 30 min. Subsequently, 3 mL trisodium citrate solution (38 mM) was dropped into the above suspension, followed by 1 min of stirring. Then, 13 mL sodium borohydride solution (0.075 wt%) was added dropwise to the reaction mixture under continuous stirring for the reduction of chlorauric acid, and maintained stirring for 15 min. The resulting suspension was centrifuged to remove the liquid supernatant. The Fe_3_O_4_/α-Fe_2_O_3_@Au MNCs were obtained by washing twice with DDW and grinding after drying.

The morphological and compositional characteristics of Fe_3_O_4_/α-Fe_2_O_3_ MHNRs and Fe_3_O_4_/α-Fe_2_O_3_@Au MNCs were analyzed using the scanning electron microscope (SEM) and transmission electron microscopy (TEM). The crystalline phase of Fe_3_O_4_/α-Fe_2_O_3_ MHNRs was identified by X-ray diffraction (XRD, Rigaku D/max 2500 PC) with Cu-Kα radiation. The magnetic measurement of both Fe_3_O_4_/α-Fe_2_O_3_ MHNRs and Fe_3_O_4_/α-Fe_2_O_3_@Au MNCs was measured using a vibrating sample magnetometer (VSM, ADE DMS-HF-4).

### 2.3 Construction and evaluation of the indicator-free electrochemical biosensor

The indicator-free Fe_3_O_4_/α-Fe_2_O_3_ MHNRs biosensor was sequentially constructed. In the first place, 15 μL of Fe_3_O_4_/α-Fe_2_O_3_@Au MNCs (20 mg/mL) were mixed with 15 μL of activated ssDNA (2 μM) and incubated at 37 °C for 3 h. In this process, ssDNA was bonded with Fe_3_O_4_/α-Fe_2_O_3_@Au MNCs to synthesize Fe_3_O_4_/α-Fe_2_O_3_@Au-ssDNA via Au-S bond. Additionally, magnetic separation was employed to remove supernatant, followed by washing with Phosphate Buffer Solution (PBS, pH 7.2) to eliminate the unbonded ssDNA. Furthermore, 50 μL Bovine Serum Albumin (BSA, 0.25%) was incubated with the resulting solid at 25 °C for 30 min to minimize nonspecific binding. Similarly, magnetic separation was used to isolate the target nanocomposites and PBS applied to remove unbound substances. In the next step, 30 μL of CYFRA 21-1 DNA solution (1 μM) was added to the product obtained from the previous step and hybridized at 65 °C for 5 min in a water bath shaker (Tm for ssDNA and CYFRA 21-1 DNA of 66.89 °C). Then, the magnetic separation and washing procedures were repeated. 30 μL of ultrapure water was added to form suspension, which of 9 μL was drop-casted onto the pretreated magnetic glassy carbon electrode (MGCE) surface and dried in 37 °C, and electrochemical measurements were subsequently performed on a CHI-760E electrochemical workstation containing 5 mM [Fe(CN)_6_]^3−/4−^ and 0.1 M KCl in ultrapure water with MGCE, Ag/AgCl electrode, and Pt electrode respectively as the working electrode, reference electrode, and the counter electrode. The feasibility of the stepwise fabrication process was systematically verified using CV, EIS, and DPV with the scan voltage of −0.1–0.7 V and scan rate of 100 mV/s for CV, the frequency range of 0.1 Hz–10 kHz and signal amplitude of 5 mV for EIS, and the measure voltage of 0.2 V (vs. Ag/AgCl) for DPV analysis.

While, unless specifically indicated, triplicate independent parallel experiments (n = 3) were carried out under identical conditions for validating the reliability of the data. The error bars were shown in the graphs correspond to standard deviation (SD) calculated from the replicates.

## 3 Results and discussion

### 3.1 Principle of the indicator-free electrochemical biosensor

**SCHEME 1:**
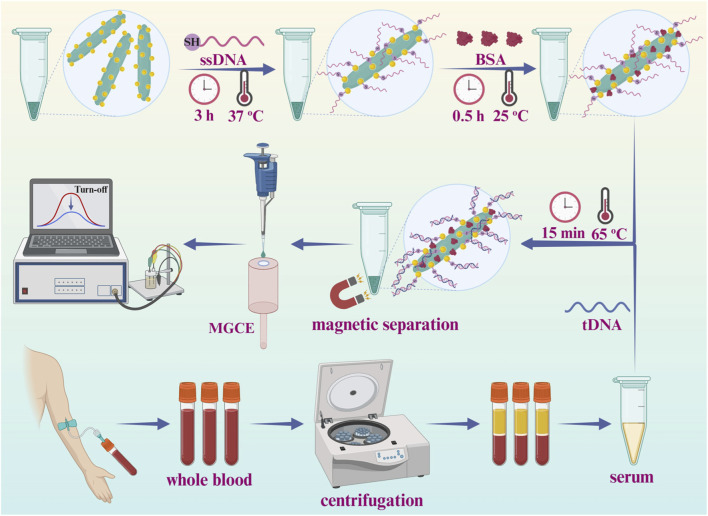
Schematic representation of the biosensor for the detection of CYFRA 21-1 DNA.

The stepwise construction procedures and detection process of the CYFRA 21-1 DNA biosensor were schematically illustrated in Scheme 1. To begin with, the integration of AuNPs with Fe_3_O_4_/α-Fe_2_O_3_ MHNRs offered abundant binding sites for ssDNA and amplified current signal. The modified ssDNA was stably immobilized onto surface Fe_3_O_4_/α-Fe_2_O_3_@Au MNCs via Au-S covalent bonding. Furthermore, BSA was employed as a blocking agent and surface passivating agent to minimize non-specific binding and prevent potential interference from extraneous factors. Lastly, the target CYFRA 21-1 DNA bound specifically to ssDNA through the principle of Watson-Crick base pairing, causing measurable alterations in electrical signals to achieve detection purposes. It was worth mentioning that in this strategy, the MHNRs could be incubated in liquid phase, and subsequently magnetically self-assembled onto the surface of MGCE, forming a uniform film to service electrochemical measurements.

### 3.2 Characteristics of nanomaterials

**FIGURE 1:**
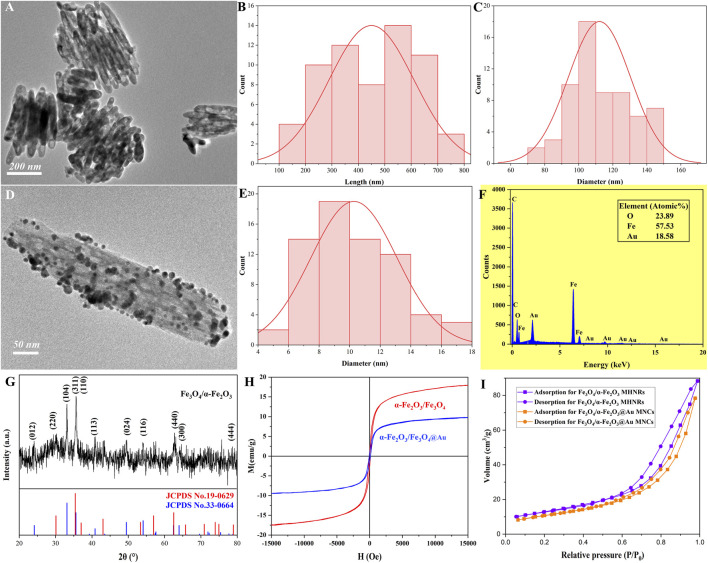
TEM image **(A)**, length distribution **(B)**, and diameter distribution **(C)** of Fe_3_O_4_/α-Fe_2_O_3_ MHNRs, TEM image **(D)** of Fe_3_O_4_/α-Fe_2_O_3_@Au MNCs, the diameter distribution **(E)** of AuNPs, EDS spectrogram **(F)** of Fe_3_O_4_/α-Fe_2_O_3_@Au MNCs, XRD pattern **(G)** of Fe_3_O_4_/α-Fe_2_O_3_ MHNRs, the hysteresis loops **(H)** and N_2_ physisorption curves **(I)** of Fe_3_O_4_/α-Fe_2_O_3_ MHNRs and Fe_3_O_4_/α-Fe_2_O_3_@Au MNCs.

The structural, morphological, and compositional properties of nanomaterials utilized to construct the biosensor were characterized, as illustrated in Figure 1. The TEM image of Fe_3_O_4_/α-Fe_2_O_3_ was presented in Figure 1A. The Fe_3_O_4_/α-Fe_2_O_3_ exhibited a well-defined rod-like morphology and uniform particle size distribution (Figures 1B,C), with an average length of 443 nm and a diameter of 109 nm. Following the reduction of HAuCl_4_ by NaBH_4_, TEM analysis (Figure 1D) revealed the successful attachment of spherical AuNPs with a diameter of 11 nm onto the surfaces of Fe_3_O_4_/α-Fe_2_O_3_ MHNRs (denoted as Fe_3_O_4_/α-Fe_2_O_3_@Au), the diameter distribution of AuNPs was shown in Figure 1E, while, the corresponding EDS spectrogram of Fe_3_O_4_/α-Fe_2_O_3_@Au MNCs was displayed in Figure 1F, the element (atomic%) ratios of O, Fe, and Au on their surfaces were 23.89%, 57.53%, and 18.58%. According to the results of EDS, the loading efficiency of AuNPs was calculated, and achieved 10.24%.

In Figure 1G, the XRD data of Fe_3_O_4_/α-Fe_2_O_3_ MHNRs was displayed. Thereinto, the diffraction peaks at 2θ = 30.1°, 35.4°, and 62.5° were indexed to the (220), (311), and (440) planes of the face-centered cubic (FCC) structure, respectively, consistent with the reference PDF card (JCPDS No. 19-0629), which indicated the existence of Fe_3_O_4_ crystalline phase. Nevertheless, the presence of distinct non-Fe_3_O_4_ characteristic diffraction peaks at 2θ = 24.1° (012), 33.2° (104), and 49.5° (024), along with an excessive peak response at approximately 35°, suggesting the existence of α-Fe_2_O_3_ in the nanomaterials, as confirmed by comparison with the reference PDF card (JCPDS No. 33-0664). The coexistence of Fe_3_O_4_ and α-Fe_2_O_3_ demonstrated the successful fabrication of Fe_3_O_4_/α-Fe_2_O_3_ MHNRs. The magnetic properties of Fe_3_O_4_/α-Fe_2_O_3_ MHNRs and Fe_3_O_4_/α-Fe_2_O_3_@Au MNCs were depicted in Figure 1H. The hysteresis loops of the two nanomaterials exhibited saturation magnetizations (*M*_*s*_) of 18.0 emu/g and 9.8 emu/g, respectively. The results revealed that following the incorporation of non-magnetic AuNPs, the *M*_*s*_ of Fe_3_O_4_/α-Fe_2_O_3_ MHNRs decreased; however, this value was sufficient for efficient magnetic separation and magnetic self-assembly procedures. N_2_ physisorption curves of Fe_3_O_4_/α-Fe_2_O_3_ MHNRs and Fe_3_O_4_/α-Fe_2_O_3_@Au MNCs were displayed in Figure 1I, their adsorption curves belonged to type-IV and H3-type hysteresis loops, and their specific surface areas reached 36.8 m^2^/g and 29.4 m^2^/g, the values were relatively smaller owing to the larger density of density. Meanwhile, the loading of AuNPs obviously led to a decrease of 7.4 m^2^/g for specific surface area, the reason for this may be that AuNPs occupied part of the pores, which also further demonstrated the successful preparation of Fe_3_O_4_/α-Fe_2_O_3_@Au MNCs.

**FIGURE 2:**
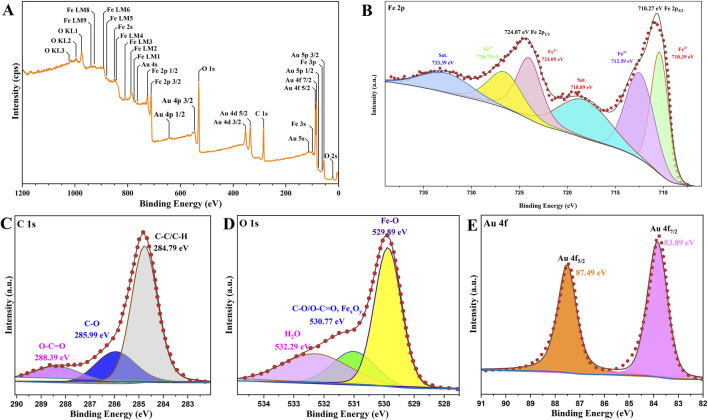
XPS survey **(A)** and Fe 2p **(B)**, C 1s **(C)**, O 1s **(D)**, and Au 4f **(E)** core-level spectra recorded of Fe_3_O_4_/α-Fe_2_O_3_@Au.

To further verify the successful preparation of Fe_3_O_4_/α-Fe_2_O_3_@Au MNCs, XPS analysis technique was employed to ascertain the chemical state and elements composition of the product as Figure 2. Firstly, the comprehensive spectrum was shown in Figure 2A, the states of all the chemical elements were annotated. Figure 2B displayed the XPS spectrum of Fe 2p, revealing the distinct peaks at 710.27 eV and 724.07 eV respectively corresponding to Fe 2p_3/2_ and Fe 2p_1/2_. The peaks at 712.59 eV and 726.79 eV indicated the existence of Fe^3+^; while, the peaks at 710.39 eV and 724.09 eV certified the existence of Fe^2+^. Furthermore, the satellite peaks resulting from energy dissipation caused by electron transitions in the valence band were observed at 718.89 eV and 733.39 eV. In the C 1s spectrum (Figure 2C), the C-O peak was observed at 285.99 eV; while, the O-C=O peak appeared at 288.39 eV. These peaks corresponded to the presence of C-O/O-C=O peak at 530.77 eV in the O 1s spectrum (Figure 2D). The spectrum of Au 4f was revealed in Figure 2E, the peaks were anastomotic with the binding energies at 83.89 eV and 87.49 eV, suggesting the existence of Au in the product. These characteristics collectively validated again the successful preparation of Fe_3_O_4_/α-Fe_2_O_3_@Au MNCs.

### 3.3 Detection feasibility of the biosensor

**FIGURE 3:**
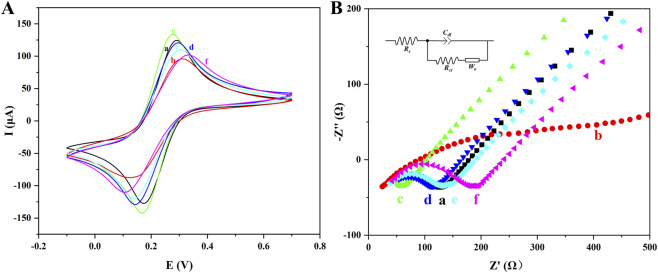
CV **(A)** and EIS spectra **(B)** of [Fe(CN)_6_]^3-/4-^ at various modified electrodes: (a) unmodified MGCE, (b) MGCE/Fe_3_O_4_/α-Fe_2_O_3_, (c) MGCE/Fe_3_O_4_/α-Fe_2_O_3_@Au, (d) MGCE/Fe_3_O_4_/α-Fe_2_O_3_@Au-ssDNA, (e) MGCE/Fe_3_O_4_/α-Fe_2_O_3_@Au-ssDNA/BSA, and (f) MGCE/Fe_3_O_4_/α-Fe_2_O_3_@Au-ssDNA/BSA/CYFRA 21-1 DNA.

The construction of the biosensor was characterized utilizing CV and EIS to validate its feasibility (Yue et al., 2024). In Figure 3A, the REDOX peaks of [Fe(CN)_6_]^3−/4−^ showed variations with different modified electrodes. Initially, compared with the current of the unmodified MGCE (curve a), the redox current significantly decreased after the modification with the Fe_3_O_4_/α-Fe_2_O_3_ MHNRs (curve b), which could be associated with the increased electron transfer resistance resulting from the MHNRs. In contrast, the current of MGCE/Fe_3_O_4_/α-Fe_2_O_3_@Au (curve c) exhibited a notable increase, which was induced by the integration of highly conductive AuNPs that evidently enhance the electron transfer rate. When the ssDNA was chemisorbed onto the MNCs through Au-S bond, the electron transfer resistance increased. Moreover, the negatively charged phosphate backbone of ssDNA introduced electrostatic repulsion with the [Fe(CN)_6_]^3−/4−^, leading to a reduction in the redox current (curve d). The current value of curve e was further reduced due to the insulating effect of BSA. Ultimately, following the material which Fe_3_O_4_/α-Fe_2_O_3_@Au-ssDNA/BSA hybridized with CYFRA 21-1 DNA was modified to the surface of MGCE, the redox current demonstrated a further reduction (curve f), the mechanism underlying current reduction was similar to the effect observed with ssDNA, indicating that ssDNA successfully captured the target CYFRA 21-1 DNA. In summary, the verification of these processes not only confirmed the feasibility of the proposed strategy but also established a robust foundation for subsequent experimental investigations.

The EIS curves which represented the electron transfer resistance (R_et_) under various loading conditions as depicted in Figure 3B. Firstly, it was essential to clarify that the semicircle radius observed in the Nyquist plot provided a direct indication of the R_et_ (Ito et al., 2019). The trends of its semicircle radius were distinctly observed in Figure 3B. With the exception of curve c, the semicircle radius of the other curves was larger compared with those of the previous steps. These results excellently agreed with the observed trends of CV curves, further indicating the detection feasibility of the biosensor.

### 3.4 Optimization of experimental conditions

The sensitivity and stability of the biosensor were critically dependent on several important experimental parameters. To achieve optimum detection performance, the following four critical parameters–the concentrations of Fe_3_O_4_/α-Fe_2_O_3_@Au MNCs and ssDNA, target hybridization time and temperature were optimized.

**FIGURE 4:**
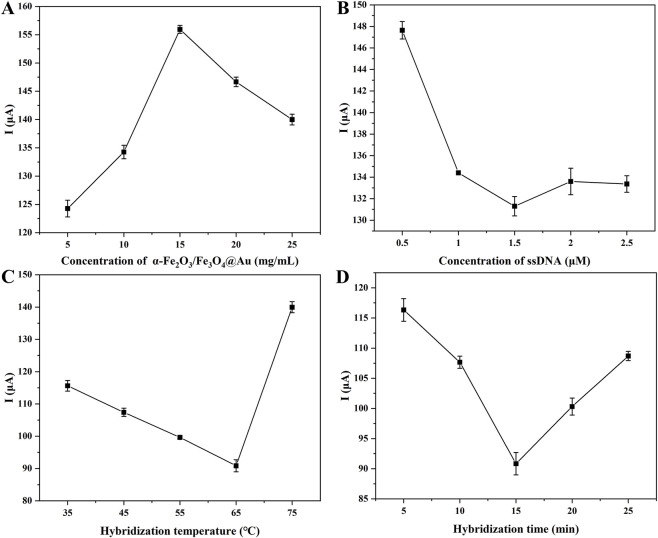
DPV responses for the optimization of the Fe_3_O_4_/α-Fe_2_O_3_@Au concentration **(A)**, the ssDNA concentration **(B)**, target hybridization time **(C)**, and target hybridization temperature **(D)**.

The quantity of Fe_3_O_4_/α-Fe_2_O_3_@Au MNCs significantly influenced the number of captured targets, the amplification effect of the current, and the incubation efficiency. Therefore, its optimization was crucial. All the results were illustrated in Figure 4A, as the concentration increased, the current values exhibited an initial rise followed by a subsequent decline. These phenomena could be induced by the insufficient current enhancement effect of AuNPs at low concentrations, whereas at high concentrations, excessive surface loading on the MGCE hindered electron transfer. Therefore, the Fe_3_O_4_/α-Fe_2_O_3_@Au MNCs with a concentration of 15 mg/mL corresponding to the highest current point was the optimal concentration.

Subsequently, the concentration optimization of ssDNA (T_m_ = 66.89 °C) was displayed in Figure 4B, which determined the maximum capture capacity of analyte. In the first place, an increase in ssDNA concentration from 0.5 μM to 1.5 μM caused a significant reduction in the current, indicating an elevation in the amount of ssDNA bound to the Fe_3_O_4_/α-Fe_2_O_3_@Au MNCs via the Au-S bond. Secondly, within the concentration range of 1.5 μM–2.5 μM, the current remained relatively stable, confirming that the available AuNPs binding sites on the MNCs were almost entirely occupied. Consequently, the results showed that the ideal ssDNA concentration was 1.5 μM, which corresponded to the current saturation point. Ultimately, optimized hybridization conditions for the capture probe and CYFRA 21-1 DNA (T_m_ = 66.89 °C) played a critical role in determining the biosensor’s sensitivity and stability. A direct correlation had been demonstrated between the number of captured CYFRA 21-1 DNA and the electrochemical response (Huang S. et al., 2024), manifested as increased R_et_ on the MCCE surface, resulting in reduced current. Hence, in Figures 4C,D, the lower current values indicating more effective probe-target complex formation. Then, the optimal hybridization conditions were determined to be 65 °C for 15 min, corresponding to the minimum current values. Following condition optimization, the biosensor’s overall performance was expected to significantly improve.

### 3.5 Detection of CYFRA 21-1 DNA

**FIGURE 5:**
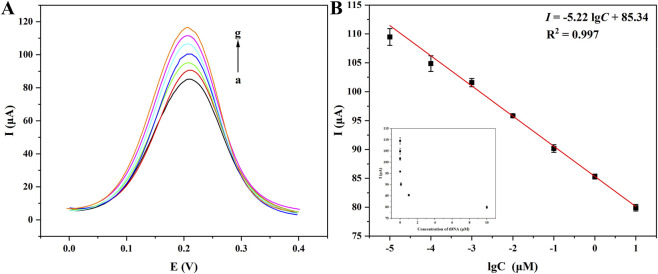
DPV curves of CYFRA 21-1 at different concentrations detected by the biosensor of Fe_3_O_4_/α-Fe_2_O_3_@Au-DNA/BSA as probe **(A)**: (a–g): 10 μM, 1 μM, 100 nM, 10 nM, 1 nM, 100 pM, 10 pM; linear relationship between different concentrations of CYFRA 21-1 DNA and currents **(B)**.

Under the optimized conditions, the biosensor’s performance was comprehensively evaluated using CYFRA21-1 DNA as the model analyte. Firstly, the correlation between CYFRA 21-1 DNA concentration (C) and the DPV current response was systematically analyzed. In Figure 5, with the increase of CYFRA 21-1 DNA concentration, the DPV peak decreased as Figure 5A, and a strong linear correlation was demonstrated between the logarithm of the CYFRA 21-1 DNA concentration and current response in the range of 10 pM–10 μM as Figure 5B. The linear equation was expressed as *I* = −5.22 lg*C* + 85.34 (*R* ^2^ = 0.997), with a limit of detection (LOD) of 1.5 pM and a limit of quantification (LOQ) of 4.5 pM, calculated by 3σ and 10σ criteria, respectively. The biosensor exhibited an extensive linear range and a low detection threshold, which could be attributed to the signal amplification capability of AuNPs and the high binding specificity of CYFRA 21-1 DNA toward ssDNA. Compared with the literature reported in past 3 years (listed in Table 1), according to the molecular weight (MW) of 196.20 for CYFRA 21-1 DNA, the liner range of this work reached 1962 pg/L-1962 μg/L (i.e. 1.962 pg/mL–1.962 μg/mL), obviously, the upper limit of the range has been largened about 20 times compared with that reported by Huang et al. (Huang Z. Q. et al., 2024), while, the lower limit of the range is only about 2 times. Therefore, the liner range has been broadened, and adapted the clinical application for the detections of healthy people and patients. While, the biosensors revealed more lower LOD, lower cost, and shorter detection period. All the data suggested their promising prospect of clinical application.

**TABLE 1:** Comparison of the reported electrochemical methods for CYFRA 21-1 detection.

Detection approach	Linear range	LOD	References
Fluorescence	0.1 fM–10^5^ fM	7.8 × 10^−2^ fM	Huang et al. (2024a)
Electrochemiluminescence	1 fg/mL–10^8^ fg/L	0.4 fg/mL	Huang et al. (2024b)
Electrochemiluminescence	10 fg/mL–5 × 10^7^ ng/mL	5.5 fg/mL	Ren et al. (2024a)
Electrochemiluminescence	10^2^ fg/mL−10^7^ fg/mL	46 fg/mL	Ren et al. (2024b)
Electrochemical	10^4^ fg/mL−10^10^ fg/mL	10^4^ fg/mL	Wang et al. (2023)
Photoelectrochemical	10^2^ fg/mL–5 × 10^7^ fg/mL	50 fg/mL	Zhang et al. (2023)
Electrochemical	15 fg/mL–90 × 10^3^ fg/mL	4.59 × 10^3^ fg/mL	Aydın et al. (2023)
Electrochemical	5 × 10^3^ fg/mL–4 × 10^8^ fg/mL	1.15 × 10^3^ fg/mL	Cai et al. (2023)
Electrochemical	10^4^ fM–10^10^ fM	1.5 × 10^3^ fM	This work

### 3.6 Selectivity, reproducibility, stability of the biosensor

**FIGURE 6:**
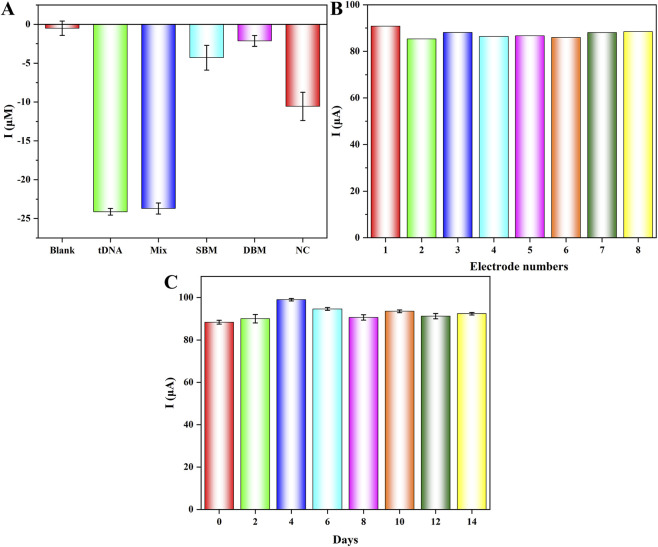
Selectivity **(A)**, reproducibility **(B)**, and stability **(C)** study of the biosensor using Fe_3_O_4_/α-Fe_2_O_3_@Au-DNA/BSA as probes.

To further evaluate the biosensor’s performance, systematic investigations were conducted to verify its selectivity, reproducibility, and stability. To begin with, comparative detection experiments were performed using SBM DNA, DBM DNA, and NC DNA as potential interfering substances to assess the selectivity and eliminate potential false positive signals, with the results compared against the CYFRA 21-1 DNA target group. As elucidated in Figure 6A, the current difference (ΔI) between the other groups (SBM DNA, DBM DNA, NC DNA, and their mixture (Mix)) and blank group was minimal, within a 5 μA margin, whereas the tDNA group revealed a distinct response with ΔI of −24.1 μA, and the Mix group also revealed similar response with ΔI of −23.7 μA, the response of Mix had no influence because of the addition of the other substances, which revealed the excellent antijamming capability of the biosensor. The experimental results conclusively demonstrated the high specificity of this biosensor.

Furthermore, under optimized and strictly controlled experimental conditions, eight parallel tDNA detection experiments were executed (Figure 6B), with a relative standard deviation (RSD) of 2.01%, confirming excellent reproducibility. Lastly, Figure 6C presented the measurement results of batch-prepared Fe_3_O_4_/α-Fe_2_O_3_@Au-ssDNA/BSA nanocomposites, which were stored in stored at 4 °C in PBS (pH = 7.4) for varying durations prior to incubation with tDNA. The measurement results revealed that the biosensor currents exhibited small fluctuation over the course of 14 days, confirming it possessed long-term storage stability. In conclusion, the developed biosensor demonstrated exceptional selectivity, reproducibility, and operational stability, rendering significant potential for practical applications in cancer diagnosis.

### 3.7 Analysis of real samples

The addition-recovery strategy with 20-fold diluted human serum was designed for evaluating the application of the biosensor in real samples. Varying concentrations of spiked CYFRA 21-1 (10 nM, 1 nM, and 100 pM) were measured. The results with treated current responses were outlined in Table 2, the recovery rates were indicated to range from 90.0% to 107.8%, and the relative standard deviation (RSD) was ≤5.2%, indicating excellent precision. In conclusion, the biosensor demonstrated outstanding resistance to interference and practical application capability, which suggesting potential for clinical detection purposes.

**TABLE 2:** Determination of CYFRA 21-1 concentration in spiked human serum samples using Fe_3_O_4_/α-Fe_2_O_3_@Au-DNA/BSA as probes.

Spiked (nM)	Test (nM)	Recovery (%)	RSD (%)
10	10.64	106.4	4.3
1	1.078	107.8	1.8
0.1	0.090	90.0	5.2

## 4 Conclusion

In this project, an indicator-free electrochemical biosensor was competently constructed, which employed the Fe_3_O_4_/α-Fe_2_O_3_ MHNRs as basic framework for supersensitive detection of CYFRA 21-1 DNA. The AuNPs-based modification strategy not only created binding sites for ssDNA, but also amplified the current signal. The target CYFRA21-1 DNA was captured through high-affinity base complementary pairing. The increase in electrochemical current verified this process and achieved the detection purpose. The optimization experiments of the construction conditions had enhanced the sensitivity of this biosensor and establishes the foundation for performance analysis. This biosensor had high specificity, which could capable distinguishing SBM sequence, DBM sequence, and NC sequence. Furthermore, it possessed excellent reproducibility, stability, and the ability to conduct real sample analysis. In the future, the platform would be expected to develop into a powerful detection methodology for cancer biomarkers and other analytes, with promising translational prospects in clinical point-of-care diagnostics.

## Data Availability

The original contributions presented in the study are included in the article/Supplementary Material, further inquiries can be directed to the corresponding authors.
