# Supplementary material for: Construction of the indicator-free electrochemical biosensor with magnetically self-assembly based on Fe3O4/α-Fe2O3 magnetic heterogeneous nanorods for the ultra-sensitive detection of CYFRA 21-1 DNA
Source: Front Chem. 2025 Dec 12;13:1696542. doi: 10.3389/fchem.2025.1696542 (PMC12740924; doi:10.3389/fchem.2025.1696542)
Supplement: Supplementary file 1 [file DataSheet1.docx]

Supplementary Material

# 2.1 Materials

All DNA sequences were supplied from Sangon Biotech Co. Ltd. (Shanghai, China). Tris-(2-carboxyethyl)-phosphine (TCEP) was obtained from Aladdin Reagent (Shanghai, China). 1× phosphate buffered saline (PBS) was ordered from Servicebio Technology Co. Ltd. (Wuhan, China). Bovine serum albumin (BSA) was ordered from Saiguo Biotech Co., Ltd. (Guangzhou, China). Polyvinylpyrrolidone (PVP), C_6_H_12_O_6_ (galactose), KCl, K_3_Fe(CN)_6_, K_4_Fe(CN)_6_·3H_2_O, FeCl_3_, NaBH_4_, HAuCl_4_·4H_2_O, and C_6_H_5_Na_3_O_7_ were purchased from Sinopharm Chemical Reagent Co., Ltd. (Shanghai, China). Polyethyleneimine (PEI) was purchased from Macklin Biochemical (Shanghai, China). Absolute ethyl alcohol was obtained from Chengdu Chron Chemicals Co., Ltd. (Sichuan, China). Human serum was provided by the Danyang People's Hospital (Zhenjiang, China).
